# What influence mothers’ mental health and health care seeking behaviors for their malnourished children in Nepal: building evidence for a broader perspective

**DOI:** 10.1192/j.eurpsy.2024.162

**Published:** 2024-08-27

**Authors:** K. Le Roch, B. Tonon, O. Acharya

**Affiliations:** ^1^Mental Health and Psychosocial Support; ^2^Nutrition and Health, Action contre la Faim, Montreuil, France; ^3^Nutrition and Health, Action contre la Faim, Kathmandu, Nepal

## Abstract

**Introduction:**

Implementing research projects on community-based health care interventions in low-ressource settings is feasible with specific methods and applications. In order to critically understand all ins and outs of influencing factors involved in health care pathways for children and their mothers, we must consider to implement more than one research in the same context.

**Objectives:**

The objective of this presentation is to showcase the continuum of research projects starting from the assessment of the effectiveness of a combined nutrition and psychosocial intervention and its economic evaluation, and how that led to exploring social representations of malnutrition in order to better understand the link with health care seeking behaviours.

**Methods:**

The FUSAM cluster randomized control trial included 427 were severe acutely malnourished (SAM) children and their mothers. They were divided in two groups receiving the standard nutrition treatment while the intervention group benefited from five psychosocial sessions. A battery of tests for child development and maternal mental health was administered pre and post intervention. For the economic evaluation, a data collection was conducted with 98 community members and District Public Health Office personnel in Saptari and 17 Action contre la Faim and government personnel in Kathmandu. Finally, a mixed-method study comparing social representations of malnutrition included 376 adults in Saptari and Nuwakot district. Data analysis was performed according to the study design: a multivariate model analysis for the CRCT, a micro-costing methodology to cost data collection and analysis was favored. For the mixed-method analysis, descriptive and inductive analysis were performed.

**Results:**

Regarding the child development, children in the intervention group showed higher scores than children in the control group at all time points. And the economic evaluation showed that the costs of adding psychosocial counselling to an existing CMAM program was approximately EUR 28,788 for 6 centers per year. However, referrals of children through the community-based screening were not optimal. The findings related to health seeking behaviors showed that different meaning categories were simultaneously resorted to by community members leading to different representations of SAM children and that relevant health advises were neither systematically nor uniquely associated to medical categories but are linked to different meaning categories depending on the cultural context.

**Conclusions:**

Multiplying research projects is crucial to mitigate the limitations of the studies often facing numerous contextual challenges and ultimately to leverage further opportunities.

**Disclosure of Interest:**

None Declared

